# GLP-1 and GIP receptor agonists in the treatment of Parkinson’s disease: Translational systematic review and meta-analysis protocol of clinical and preclinical studies

**DOI:** 10.1371/journal.pone.0255726

**Published:** 2021-08-12

**Authors:** Carolina Vaccari, Denise Grotto, Tiago da V. Pereira, João Lauro V. de Camargo, Luciane C. Lopes

**Affiliations:** 1 Department of Pathology, São Paulo State University (UNESP), Botucatu, São Paulo, Brazil; 2 Graduate Course of Pharmaceutical Sciences, University of Sorocaba (UNISO), Sorocaba, São Paulo, Brazil; 3 Applied Health Research Centre, St. Michael’s Hospital, University of Toronto, Toronto, Canada; 4 Department of Health Sciences, University of Leicester, Leicester, United Kingdom; Universiti Tunku Abdul Rahman, MALAYSIA

## Abstract

**Background:**

Parkinson’s disease (PD) is a progressive multifactorial neurodegenerative condition. Epidemiological studies have shown that patients with type 2 diabetes mellitus (T2DM2) are at increased risk for developing PD, indicating a possible insulin-modulating role in this latter condition. We hypothesized that drugs similar to glucagon-like peptide-1 (GLP-1) and gastric inhibitory polypeptide (GIP), used in the treatment of T2DM2, may play a role in PD.

**Objectives:**

The purpose of this study is to systematically review and meta-analyze data of preclinical and clinical studies evaluating the efficacy and safety of GLP-1 and GIP drugs in the treatment of PD.

**Methods:**

Two reviewers will independently evaluate the studies available in the Ovid Medline, Ovid Embase, Web of Science, Cochrane Central Register of Controlled Trials, Cinahl, and Lilacs databases. Preclinical rodent or non-human primate studies and randomized controlled human clinical trials will be included, without language or publication period restrictions. Outcomes of interest in preclinical studies will be primarily locomotor improvements and adverse effects in animal models of PD. For clinical trials, we will evaluate clinical improvements rated by the Movement Disorders Society Unified Parkinson’s Disease Rating Scale–parts I, II, III, and IV, and adverse effects. The risk of bias of preclinical studies will be assessed by the SYRCLE tool and CAMARADES checklist and the clinical studies by the Cochrane tool; the certainty of the evidence will be rated by GRADE.

**Discussion and conclusion:**

There is an urge for new PD treatments that may slow the progression of the disease rather than just restoring dopamine levels. This study will comprehensively review and update the state of the art of what is known about incretin hormones and PD and highlight the strengths and limitations of translating preclinical data to the clinic whenever possible.

**Systematic review registration:**

PROSPERO registration number CRD42020223435.

## Background

Glucagon-like peptide-1 (GLP-1) and glucose-dependent insulinotropic polypeptide (GIP) are incretin hormones secreted by specific intestinal enteroendocrine cells in response to nutrient ingestion and absorption, preferably of carbohydrate and fat [1–3].

Both hormone effects are characterized by stimulation of pancreatic islet β cell proliferation and differentiation, as well as induction of glucose-dependent pancreatic insulin secretion [2, 4]. Curiously, GLP-1 also acts as a potent glucagon inhibitor in a postprandial state, while GIP stimulates glucagon release during fasting [2, 5, 6]. Together, they play an important role in regulating glycemic homeostasis, and, for that reason, analogs have been used in the treatment of type II diabetes mellitus (T2DM) [7, 8].

In addition to their conventional application in the T2DM treatment, several *in vitro* and *in vivo* studies [9–14] have evaluated the GLP-1 and GIP pleiotropic actions in extrapancreatic tissues, such as neuroprotective and neurotrophic effects in Parkinson’s disease (PD) models.

PD is a chronic and progressive neurodegenerative disorder characterized by both motor and non-motor symptoms [15]. The pathological selective degeneration of dopaminergic neurons in one of the basal ganglia termed *substantia nigra pars compacta* (SNpc) culminates with reduced dopamine production and motor impairment. One of the morphological hallmarks of PD is the intracytoplasmic fibrillar aggregates referred to as Lewy bodies, in which the protein α-synuclein is a major component [15]. The causes of PD are not well defined. However, it is suggested that its pathogenesis is multifactorial, involving genetic susceptibility, aging and exposure to certain chemical agents [16].

T2DM has been associated with more severe symptoms and accelerated progression of PD [17–19]. The importance of metabolic dysfunction in PD is becoming widely accepted, including dysfunction of insulin signaling pathways [17, 20, 21].

In the CNS, insulin is behind many processes that could be dysregulated in PD, including apoptosis, autophagy, mitochondrial dysfunction, oxidative stress, neuroinflammation, and synaptic plasticity [22–27]. It is even possible that there is a relationship between the accumulation of the anomalous α-synuclein protein and the development of insulin resistance [22]. In transgenic mice with overexpression of alpha-synuclein, the accumulation of this protein appears to act as a negative regulator of the insulin signaling pathway by destabilizing its receptor substrate (Insulin receptor substrate-1, IRS-1) and inhibiting activation of the other important protein kinases of the pathway [28]. This may trigger a vicious cycle of neuroinflammation and worsen the α-synuclein accumulation, which could lead to more neuronal loss.

Animal studies have shown that obese rats [29] or mice [30] or transgenic mice [31] with a diabetic profile are more susceptible to the neurotoxins used in PD animal models, such as by the development of brain insulin resistance [29, 31], accumulation of intracytoplasmic α-synuclein protein in the SNpc [31], activation of microglia cells (neuroinflammation) [31], endoplasmic reticulum stress in the midbrain [31], dopamine depletion in the SNpc and striatum [29], oxidative stress in dopaminergic neurons [29, 30] and/or dopaminergic neuronal loss [31].

Apart from peripheral tissues, the incretin hormone receptors are also expressed in the CNS [32–35]. The binding to GLP-1 and GIP receptors by GLP-1 and GIP agonists may restore brain insulin sensitivity by activation of specific insulin-modulated pathways that promote cell survival, while inhibiting pro-apoptotic pathways [36–39]. Therefore, interventions aimed at reversing insulin resistance are being increasingly recognized as possible new therapies for PD.

However, the GLP-1 and GIP receptor agonists may differ in their neuroprotective effects in view of pharmacodynamic and pharmacokinetic differences, and it is not quite clear if they would act as adjuvants in the treatment of PD with other usual antiparkinsonian drugs, or if they really could alter the progression of the disease.

Studies comparing different animal models of PD and different experimental designs with incretin hormones are scarce [40]. Either, there are a few randomized controlled trials with these analogs available in the literature; therefore, the possibility of translating the efficacy of this group of drugs from preclinical studies to the clinic has not yet been evaluated.

Translating the knowledge of basic research into new therapeutic approaches following the concept of "bench to bedside" is just one of the stages of translational medical research [41, 42]. Therefore, we propose to perform a systematic review and meta-analysis of randomized controlled clinical trials and preclinical studies evaluating the efficacy and safety of GLP-1 and GIP receptor agonists in slowing the progression of PD, exploring their potential mechanisms of neuroprotection. By analyzing preclinical and clinical studies, the ultimate goal is to verify if the results from preclinical studies can be translated into clinical practice whenever possible.

## Methods

### Standards

This protocol will follow established guidelines for systematic reviews and meta-analyses of clinical trials (PRISMA-P) [43], and specific guidelines for conducting systematic reviews of preclinical studies [44].

### Protocol registration number

This protocol is registered in the International Prospective Register of Systematic Reviews (PROSPERO registration number: CRD42020223435).

### Eligibility criteria

#### For clinical studies

*Study design*. Randomized controlled trials.

*Participants*. Studies including adults (≥ 18 years old) with a specialist-confirmed PD diagnosis (ICD-10: G20) according to the International Parkinson and Movement Disorder Society (MDS) criteria or the UK Parkinson’s Disease Society Brain Bank diagnostic criteria, or on the basis of clinical neurological assessment [45]. There will be no restrictions regarding sex, ethnicity or severity of PD.

*Intervention*. Studies including patients on treatment of PD with drugs capable of activating the following receptors:

- GLP-1: GLP-1, liraglutide, lixisenatide, semaglutide, geniposide, exenatide, exendin-4, oxytomodulin or others;
- GIP: D-Ala2-GIP-glu-PAL or others;
- GLP-1/GIP (dual agonists): DA-JC1, DA-JC4, DA-CH5, DA3-CH or others.

There will be no restrictions regarding duration and doses. Patients concomitantly receiving usual antiparkinsonian drugs (e.g., levodopa or dopaminergic drugs, such as monoamine oxidase inhibitors, catechol-O-methyltransferase inhibitors or dopaminergic agonists) will be accepted.

*Comparators*. Studies including specialist-confirmed PD patients who have not received treatment with GLP-1, GIP or dual GLP-1/GIP receptor agonist drugs. Participants on GLP-1/GIP placebos receiving standard of care (e.g., background treatment with antiparkinsonian drugs) will also be accepted.

*Primary outcomes*. Studies that measured:

- PD motor signs rated by the “Movement Disorders Society Unified Parkinson’s Disease Rating Scale—part III [46]”. A difference of -3.25 points will be considered a minimal, but clinically important, improvement and 4.63 points will be considered minimal, but clinically important, worsening [47].
- Non-motor symptoms rated by the MDS-UPDRS Scale, part I [46] or the Non-Motor Symptoms Questionnaire [48];
- Activities of daily living rated by the MDS-UPDRS Scale, part II [46];
- Dyskinesias or motor fluctuations rated by the MDS-UPDRS Scale part IV [46] or the Unified Dyskinesia Rating Scale [49];
- Adverse effects or serious adverse effects as reported in the included studies.

*Secondary outcomes*. Studies that measured:

- Quality of life rated by validated scales or questionnaires, such as the Parkinson’s Disease 39 item Quality of life questionnaire [50], the Parkinson’s disease quality of life questionnaire [51] or others;
- Psychological alterations, such as depression or dementia rated by the Mattis Dementia Rating Scale [52] or the Montgomery-Asberg Depression Rating Scale [53].

*Exclusion criteria*. Considering the potential association between diabetes and PD, we will exclude studies that exclusively enrolled patients with diabetes (any type). Furthermore, we will exclude studies involving participants with advanced dementia because cognitive impairment could affect the accuracy of patient-reported outcomes.

#### For preclinical studies

*Study design*. Any non-human studies using the following most common neurotoxin-induced animal models of PD for the establishment of therapeutic measures:

- 6-hydroxydopamine,1-methyl-4-phenyl-1,2,3,6-tetrahydropyridine, lipopolysaccharide, rotenone (or other possible pesticide exposure);
- Transgenic animal models that explore the function of PD-linked genes (e.g. α-synuclein, DJ-1, LRRK2, Parkin, UCH-L1, PINK1);
- Other models mentioned in the screened studies.

*Animal species*. Studies with adult rodents or non-human primates, without restriction as to strain, sex, life stage of exposure and/or moment of outcome evaluation.

*Intervention*. Studies including PD treatment with drugs capable of activating the following receptors:

- GLP-1: GLP-1, liraglutide, lixisenatide, semaglutide, geniposide, exenatide, exendin-4, oxytomodulin or others;
- GIP: D-Ala2-GIP-glu-PAL or others;
- GLP-1/GIP (dual agonists): DA-JC1, DA-JC4, DA-CH5, DA3-CH or others receptor agonist drugs.

Only studies that performed exposure to each drug separately will be accepted. There will be no restriction on dose level, route of administration or duration of exposure. Animals concomitantly treated with usual antiparkinsonian drugs will be accepted.

*Comparators*. Studies including PD or vehicle animals not exposed to the GLP-1, GIP or GLP-1/GIP receptor agonist drugs. Control animals treated with usual antiparkinsonian drugs will be accepted.

*Primary outcomes*. Studies that measured:

- Motor signs (motor activity and sensorimotor reflex) in tests commonly used to assess neurological parameters in rodents/non-human primates;
- Mortality or adverse effects, such as body weight changes, organ weight changes, macroscopical and histopathological analysis, liver or pancreatic enzyme levels, glucose levels, or others as reported in the included studies.

*Secondary outcomes*. Studies that measured cognitive functions/neurological behaviors and/or morphological changes that could be indicative of the GLP-1/GIP mode of action, such as:

- Learning and memory in tests commonly used to assess neurological parameters in rodents/non-human primates;
- Anxiety or depression behaviors measured by tests commonly used to assess neurological parameters in rodents/non-human primates;
- Number of nigrostriatal dopaminergic neurons by positive immunostaining of the tyrosine hydroxylase enzyme of neurons in the SNpc and other CNS regions;
- Quantification of dopamine levels and its metabolites in the striated nucleus or other CNS regions;
- Quantification of antiapoptotic signaling molecules;
- Evaluation of neuroinflammation by quantifying inflammatory cytokines or transcription factors involved in inflammatory response;
- α-synuclein accumulation in the SNpc and other CNS regions.
- Quantification of reactive oxygen species in dopaminergic neurons, as well as quantification of markers for oxidative stress and antioxidant enzymes;
- Quantification of neurotrophic factors related to neuroplasticity mechanisms.

### Search methods for primary studies

#### Electronic searches

Peer-reviewed original studies published in the following electronic databases will be searched: Ovid Medline, Ovid Embase, Web of Science, Cochrane Central Register of Controlled Trials, Cinahl, and Lilacs without language and year of publication restrictions. Clinicaltrials.gov will also be consulted especially concerning possible unpublished trials with available data. All electronic searches will be performed from database inception to December, 2021.

#### Search strategy

Key terms, MeSH terms and free terms related to “Parkinson’s Disease”, “Parkinsonian Disorders”, “Glucagon-Like Peptide 1”, “Glucagon-Like Peptide 1 receptor”, “Exenatide”, “Liraglutide”, “Lixisenatide”, “Semaglutide”, “Geniposide”, “Oxytomodulin”, “D-Ala2-GIP-glu-PAL”, “DA-JC1”, “DA-JC4”, “DA-CH5”, “DA3-CH”, and “Glucose-dependent insulinotropic polypeptide” will be combined for each database (see S1 Appendix for an example of search strategy that will be adapted for each database).

#### Searching other resources

References listed in the selected studies will be analyzed for additional citations. Corresponding authors will be contacted to retrieve additional data.

### Eligibility determination

Two reviewers (CV and DG), after calibration exercises, will independently screen all titles and abstracts identified by the literature search, obtain full-text articles of all potentially eligible studies, and evaluate them for eligibility. Disagreements will be resolved by consensus and, eventually, through consultation with technical advisors (JLVC, LCL, TVP) to improve accuracy and consistency among screeners.

### Study flow diagram

A PRISMA flow diagram will be performed to indicate the number of included and excluded studies and the corresponding reasons for exclusion.

### Data extraction

Reviewers (CV and DG) will undergo calibration exercises, and work in pairs to independently extract data from the included studies. A standardized, piloted form will be used to extract information from the clinical studies: reference, name of the evaluated drug, study design, severity of PD, name of the antiparkinsonian drugs possibly used concomitantly with the incretin analogs, treatment characteristics (dose, period of application, interval between applications, number of applications) and presentation of results (outcomes and the respective evaluation methods in the control and treatment groups).

For preclinical studies, the following information will be extracted: reference, name of the evaluated drug, description of the PD animal model (including strain, age, sex, the weight of animals and name of the PD-inducing neurotoxin or transgenic model), type and source of food used, conditions of the housing, control of temperature and humidity, characteristics of the test substance, experimental design (number of animals/group, acute or chronic exposure), treatment (doses, number of applications, vehicle, route of administration), euthanasia protocol, anesthetics possibly used, results (outcomes and the respective evaluation methods for control and treatment groups), and description of the statistics used. Whenever results in the preclinical studies are presented in graphs, the measures will be estimated by the Digitzeit software (version 2.4) in pairs, independently (CV and DG), and the mean of both measures will be extracted in case of minor divergence. Disagreements will be resolved by consensus and, eventually, through consultation with technical advisors (JLVC, LCL, TVP) to improve accuracy and consistency among screeners.

### Assessment of internal validity of individual studies

Two reviewers (CV and DG) will independently assess the internal validity of each study. Disagreements will be resolved by consensus and, eventually, through consultation with technical advisors.

For clinical studies, the Cochrane risk of bias tool will be used to assess the risk of bias [54, 55]. The following domains will be considered: a) selection bias (random sequence generation/allocation concealment); b) performance bias (blinding of participants and personnel); c) detection bias (blinding of outcome assessment); d) attrition bias (incomplete outcome data); e) other sources of bias.

For preclinical studies, specific assessment tools will be applied to evaluate the risk of bias, including the SYRCLE risk of bias tool [56] and the CAMARADES checklist [57], respectively.

The following domains will be considered for the risk of bias assessment: a) selection bias (random sequence generation/baseline characteristics/allocation concealment); b) performance bias (random housing/blinding of researchers during intervention); c) detection bias (random outcome assessment/outcome assessment blinding); d) attrition bias (incomplete outcome data); e) other sources of bias.

The possible answers for each domain in the risk of bias assessment of clinical and preclinical studies will be “Yes” (high risk of bias), “No” (low risk of bias) or “Unclear” (unclear risk of bias). Results will be presented in the form of tables and graphs.

### Data synthesis and statistical analysis

Meta-analyses will be performed separately for preclinical and clinical studies, for each outcome (primary and secondary outcomes) through an inverse-variance random effects model. We will use the restricted maximum-likelihood (REML) estimator of the between-study variance. However, we will also present the results for the inverse-variance fixed-effects model as a sensitivity analysis.

Whenever feasible, inter-species comparison analyses will be performed to contrast the treatment effects observed in animal studies to those detected in human trials (e.g., a drug’s effect on motor function in animals will be compared to the corresponding effects on humans). Inter-species comparisons will consider the direction, magnitude, and uncertainty of effects. Morphological outcomes in preclinical studies will be used to elucidate the GLP-1/GIP possible neuroprotective mode of action and will be in separate meta-analyses whenever possible or summarized in descriptive tables. All meta-analyses will be performed by the Stata software package (version 14.2) and presented in forest plots.

To perform the meta-analyses, the same measure of association will be calculated for the studies, depending on the type of data:

- Dichotomous outcome—Relative risks will be used as the effect measure, with a 95% confidence interval.
- Continuous outcome—Mean differences (MD) or standardized mean differences (SMD) will be used as the measure of treatment effect, with a 95% confidence interval.

#### Funnel plot asymmetry

Funnel plot asymmetry will be limited to outcomes with 10 or more estimates. We will plot a measure of precision (e.g., standard error) on the vertical axis and the corresponding estimate on the horizontal axis. We will also use Egger’s regression test for continuous outcomes and Harbord’s test for binary outcomes [58, 59].

#### Heterogeneity assessment

Heterogeneity among individual studies will be assessed using the I^2^ statistic and Cochran’s Q test [60]. The latter will be considered statistically significant when P < 0.10.

#### Meta-regression and subgroup analysis

Meta-regression models with the REML estimator of the between-study variance will be fitted whenever 10 or more estimates are available. If less than 10 estimates are available, subgroup analyses will be performed. Meta-regression and subgroup analyses will be conducted whenever possible (at least 2 studies) to investigate possible sources of heterogeneity in clinical studies, such as due to the inclusion of participants with different PD severity (severe, moderate and mild scored on the MDS-UPDRS part III), subtypes of PD (tremor dominant, mixed and akinetic/rigid), genomic status of each patient’s PD related genes (α-synuclein, DJ-1, LRRK2, Parkin, UCH-L1 or PINK1), use of other antiparkinsonian drugs (levodopa or dopaminergic drugs, such as monoamine oxidase inhibitors, catechol-O-methyltransferase inhibitors or dopaminergic agonists), and different treatment schemes with the GLP-1, GIP or GLP-1/GIP receptor agonists (different doses, route of administration and duration of treatment). In case of concomitant use with other antiparkinsonian drugs, we will consider whether the patients were in an “off” medication state (with the withdrawal of the medication at least the day before the evaluations). Whenever possible, “on” and “off” state measures will be compared separately since it is expected a higher magnitude of effect in an “on” medication state.

For preclinical studies, heterogeneity will be investigated considering the use of different animal species (rats, mice and non-human primates), different PD models (different neurotoxins and transgenic models), different PD severity (partial and full lesions), use of other antiparkinsonian drugs (levodopa or dopaminergic drugs, such as monoamine oxidase inhibitors, catechol-O-methyltransferase inhibitors or dopaminergic agonists) and different treatment schemes (different doses, route of administration and duration of treatment).

If there are enough studies (at least 2), we will investigate the impact of high risk of bias studies on the meta-analyses in a sensitivity analysis.

If it is not possible to pool results in the meta-analyses due to high not explained heterogeneity or not enough similar data for each outcome, findings will only be narratively described.

### Certainty of evidence

The GRADE system [61–63] will be used to rate the certainty of evidence provided by preclinical and clinical studies separately. The certainty of evidence will be determined for each outcome. Randomized controlled clinical trials and animal studies, when well designed, can eliminate much of the confounding factors by random allocation of participants and animals to the different study groups. Thus, they start from the initial classification of "high certainty".

However, there are several factors that can downgrade the initial classification of the study groups in relation to the reliability of the evidence that they generated: risk of bias [64], imprecision [65], inconsistency [66], indirectness [67], and publication bias [68].

Four descriptors will be used to indicate the level of confidence in the evidence generated by preclinical and clinical studies, separately: high certainty level (++++); moderate certainty level (+++); low certainty level (++); very low certainty level (+). All results will be presented as tables.

Highly reliable evidence implies that further studies will hardly change the available results; on the other hand, very low levels of evidence indicate that future studies may alter not only the confidence in the available results, but the results themselves.

## Discussion and conclusion

Ever since its discovery in the late 60s, levodopa remains the gold standard for the symptomatic treatment of PD [69]. Levodopa is a dopamine precursor that easily crosses the blood-brain barrier, and in the CNS is converted to dopamine to restore the low dopamine levels caused by dopaminergic neuron degeneration. However, long-term levodopa treatment is frequently associated with motor fluctuations and dyskinesias that have a serious impact on patient quality of life [69].

Currently, the goal for the treatment of PD is the development of drugs that may slow the progression of the disease, instead of just restoring dopamine levels. GLP-1 and GIP receptor agonists have been indicated as possible neuroprotectors due to a reduction in neuronal insulin resistance in a variety of preclinical models [22, 36–38].

The use of animal models has been crucial to elucidate the pathophysiology behind PD and the development of therapeutic strategies. However, each model has its own particularities and there is still no single model that mimics all the human features of PD [70, 71].

Although the clinical aspects in animals are not the same as in humans, there are different locomotor tests that investigate bradykinesia, decreased motor activity, tremors, muscle rigidity or motor coordination in animals, besides neurobehavioral conditions, such as anxiety, depression, memory loss and others [71]. In the present work, corresponding outcomes will be used for more straightforward comparisons with clinical findings in humans whenever possible. The morphological outcomes in animals will be used to investigate the mechanisms of action in both animals and humans. Naturally, the future discussion will also take into account the applicability and limitations of the current PD animal models.

It is acknowledged that, as each outcome will be evaluated separately, meta-analyses and subgroup analyses eventually will not be possible due to the lack of studies with similar data or due to high heterogeneous findings. It is also possible to predict a low number of clinical trials that do not address all drugs identified in preclinical studies.

Nevertheless, this study will update the state of the art in terms of what is known about incretin hormones and PD and provide a critical appraisal on the possible efficacy and safety of these drugs in altering the course of the disease, highlighting the strengths and limitations of translating preclinical data to the clinic whenever possible.

## Supporting information

S1 Checklist PRISMA-P (Preferred Reporting Items for Systematic review and Meta-Analysis Protocols) 2015 checklist: Recommended items to address in a systematic review protocol*. (DOC)

S1 Appendix Search strategy OVID MEDLINE. Search strategy example to be adapted for each database.
(DOCX)

## References

[1] Drucker DJ. The biology of incretin hormones. Cell metabolism. 2006;3(3):153–65. Epub 2006/03/07. doi: 10.1016/j.cmet.2006.01.004 . 16517403

[2] Nauck MA, Meier JJ. Incretin hormones: Their role in health and disease. Diabetes, obesity & metabolism. 2018;20 Suppl 1:5–21. Epub 2018/01/25. doi: 10.1111/dom.13129 . 29364588

[3] Wu T, Rayner CK, Horowitz M. Incretins. Handbook of experimental pharmacology. 2016;233:137–71. Epub 2015/04/24. doi: 10.1007/164_2015_9 . 25903418

[4] Baggio LL, Drucker DJ. Biology of incretins: GLP-1 and GIP. Gastroenterology. 2007;132(6):2131–57. Epub 2007/05/15. doi: 10.1053/j.gastro.2007.03.054 . 17498508

[5] Holst JJ. The physiology of glucagon-like peptide 1. Physiological reviews. 2007;87(4):1409–39. Epub 2007/10/12. doi: 10.1152/physrev.00034.2006 . 17928588

[6] Meier JJ, Gallwitz B, Siepmann N, Holst JJ, Deacon CF, Schmidt WE, et al. Gastric inhibitory polypeptide (GIP) dose-dependently stimulates glucagon secretion in healthy human subjects at euglycaemia. Diabetologia. 2003;46(6):798–801. Epub 2003/05/24. doi: 10.1007/s00125-003-1103-y . 12764578

[7] Bailey CJ. GIP analogues and the treatment of obesity-diabetes. Peptides. 2020;125:170202. Epub 2019/11/23. doi: 10.1016/j.peptides.2019.170202. 31756366

[8] Maselli DB, Camilleri M. Effects of GLP-1 and Its Analogs on Gastric Physiology in Diabetes Mellitus and Obesity. Advances in experimental medicine and biology. 2020. Epub 2020/02/23. doi: 10.1007/5584_2020_496. 32077010

[9] Bertilsson G, Patrone C, Zachrisson O, Andersson A, Dannaeus K, Heidrich J, et al. Peptide hormone exendin-4 stimulates subventricular zone neurogenesis in the adult rodent brain and induces recovery in an animal model of Parkinson’s disease. Journal of neuroscience research. 2008;86(2):326–38. Epub 2007/09/07. doi: 10.1002/jnr.21483 . 17803225

[10] Hansen HH, Fabricius K, Barkholt P, Mikkelsen JD, Jelsing J, Pyke C, et al. Characterization of liraglutide, a glucagon-like peptide-1 (GLP-1) receptor agonist, in rat partial and full nigral 6-hydroxydopamine lesion models of Parkinson’s disease. Brain research. 2016;1646:354–65. Epub 2016/05/29. doi: 10.1016/j.brainres.2016.05.038 . 27233809

[11] Harkavyi A, Abuirmeileh A, Lever R, Kingsbury AE, Biggs CS, Whitton PS. Glucagon-like peptide 1 receptor stimulation reverses key deficits in distinct rodent models of Parkinson’s disease. Journal of neuroinflammation. 2008;5:19. Epub 2008/05/22. doi: 10.1186/1742-2094-5-19; PubMed Central PMCID: PMC2426681. 18492290 PMC2426681

[12] Li Y, Liu W, Li L, Hölscher C. D-Ala2-GIP-glu-PAL is neuroprotective in a chronic Parkinson’s disease mouse model and increases BNDF expression while reducing neuroinflammation and lipid peroxidation. European journal of pharmacology. 2017;797:162–72. Epub 2016/12/04. doi: 10.1016/j.ejphar.2016.11.050 . 27913104

[13] Zhang L, Zhang L, Li Y, Li L, Melchiorsen JU, Rosenkilde M, et al. The Novel Dual GLP-1/GIP Receptor Agonist DA-CH5 Is Superior to Single GLP-1 Receptor Agonists in the MPTP Model of Parkinson’s Disease. Journal of Parkinson’s disease. 2020. Epub 2020/01/21. doi: 10.3233/JPD-191768. 31958096

[14] Zhang ZQ, Hölscher C. GIP has neuroprotective effects in Alzheimer and Parkinson’s disease models. Peptides. 2020;125:170184. Epub 2019/11/11. doi: 10.1016/j.peptides.2019.170184. 31705913

[15] Beitz JM. Parkinson’s disease: a review. Frontiers in bioscience (Scholar edition). 2014;6:65–74. Epub 2014/01/07. doi: 10.2741/s415 . 24389262

[16] Warner TT, Schapira AH. Genetic and environmental factors in the cause of Parkinson’s disease. Annals of neurology. 2003;53 Suppl 3:S16–23; discussion S-5. Epub 2003/04/01. doi: 10.1002/ana.10487 . 12666095

[17] Camargo Maluf F, Feder D, Alves de Siqueira Carvalho A. Analysis of the Relationship between Type II Diabetes Mellitus and Parkinson’s Disease: A Systematic Review. Parkinsons Dis. 2019;2019:4951379–. doi: 10.1155/2019/4951379 . 31871617 PMC6906831

[18] Cereda E, Barichella M, Cassani E, Caccialanza R, Pezzoli G. Clinical features of Parkinson disease when onset of diabetes came first: A case-control study. Neurology. 2012;78(19):1507–11. Epub 2012/04/28. doi: 10.1212/WNL.0b013e3182553cc9 . 22539572

[19] Giuntini M, Baldacci F, Del Prete E, Bonuccelli U, Ceravolo R. Diabetes is associated with postural and cognitive domains in Parkinson’s disease. Results from a single-center study. Parkinsonism & related disorders. 2014;20(6):671–2. Epub 2014/04/02. doi: 10.1016/j.parkreldis.2014.02.016 . 24685342

[20] Arvanitakis Z, Wilson RS, Bienias JL, Bennett DA. Diabetes and parkinsonian signs in older persons. Alzheimer disease and associated disorders. 2007;21(2):144–9. Epub 2007/06/05. doi: 10.1097/WAD.0b013e31805ba768 . 17545740

[21] De Pablo-Fernandez E, Breen DP, Bouloux PM, Barker RA, Foltynie T, Warner TT. Neuroendocrine abnormalities in Parkinson’s disease. Journal of neurology, neurosurgery, and psychiatry. 2017;88(2):176–85. Epub 2016/11/02. doi: 10.1136/jnnp-2016-314601 . 27799297

[22] Athauda D, Foltynie T. The glucagon-like peptide 1 (GLP) receptor as a therapeutic target in Parkinson’s disease: mechanisms of action. Drug discovery today. 2016;21(5):802–18. Epub 2016/02/07. doi: 10.1016/j.drudis.2016.01.013 . 26851597

[23] Hirsch EC, Jenner P, Przedborski S. Pathogenesis of Parkinson’s disease. Movement disorders: official journal of the Movement Disorder Society. 2013;28(1):24–30. Epub 2012/08/29. doi: 10.1002/mds.25032 . 22927094

[24] Dyer AH, Vahdatpour C, Sanfeliu A, Tropea D. The role of Insulin-Like Growth Factor 1 (IGF-1) in brain development, maturation and neuroplasticity. Neuroscience. 2016;325:89–99. Epub 2016/04/04. doi: 10.1016/j.neuroscience.2016.03.056 . 27038749

[25] Gonzalez-Franquesa A, Patti ME. Insulin Resistance and Mitochondrial Dysfunction. Advances in experimental medicine and biology. 2017;982:465–520. Epub 2017/05/30. doi: 10.1007/978-3-319-55330-6_25 . 28551803

[26] Labandeira-Garcia JL, Costa-Besada MA, Labandeira CM, Villar-Cheda B, Rodriguez-Perez AI. Insulin-Like Growth Factor-1 and Neuroinflammation. Frontiers in aging neuroscience. 2017;9:365. Epub 2017/11/23. doi: 10.3389/fnagi.2017.00365; PubMed Central PMCID: PMC5675852. 29163145 PMC5675852

[27] Maciejczyk M, Zebrowska E, Chabowski A. Insulin Resistance and Oxidative Stress in the Brain: What’s New? International journal of molecular sciences. 2019;20(4). Epub 2019/02/20. doi: 10.3390/ijms20040874; PubMed Central PMCID: PMC6413037. 30781611 PMC6413037

[28] Gao S, Duan C, Gao G, Wang X, Yang H. Alpha-synuclein overexpression negatively regulates insulin receptor substrate 1 by activating mTORC1/S6K1 signaling. The international journal of biochemistry & cell biology. 2015;64:25–33. Epub 2015/03/31. doi: 10.1016/j.biocel.2015.03.006 . 25813876

[29] Morris JK, Bomhoff GL, Stanford JA, Geiger PC. Neurodegeneration in an animal model of Parkinson’s disease is exacerbated by a high-fat diet. Am J Physiol Regul Integr Comp Physiol. 2010;299(4):R1082–R90. Epub 2010/08/11. doi: 10.1152/ajpregu.00449.2010 . 20702796 PMC2957375

[30] Choi JY, Jang EH, Park CS, Kang JH. Enhanced susceptibility to 1-methyl-4-phenyl-1,2,3,6-tetrahydropyridine neurotoxicity in high-fat diet-induced obesity. Free radical biology & medicine. 2005;38(6):806–16. Epub 2005/02/22. doi: 10.1016/j.freeradbiomed.2004.12.008 . 15721991

[31] Wang L, Zhai YQ, Xu LL, Qiao C, Sun XL, Ding JH, et al. Metabolic inflammation exacerbates dopaminergic neuronal degeneration in response to acute MPTP challenge in type 2 diabetes mice. Experimental neurology. 2014;251:22–9. Epub 2013/11/14. doi: 10.1016/j.expneurol.2013.11.001 . 24220636

[32] Baggio LL, Drucker DJ. Glucagon-like peptide-1 receptors in the brain: controlling food intake and body weight. The Journal of clinical investigation. 2014;124(10):4223–6. Epub 2014/09/10. doi: 10.1172/JCI78371 ; PubMed Central PMCID: PMC4191040. 25202976 PMC4191040

[33] Hamilton A, Holscher C. Receptors for the incretin glucagon-like peptide-1 are expressed on neurons in the central nervous system. Neuroreport. 2009;20(13):1161–6. Epub 2009/07/21. doi: 10.1097/WNR.0b013e32832fbf14 . 19617854

[34] Nyberg J, Jacobsson C, Anderson MF, Eriksson PS. Immunohistochemical distribution of glucose-dependent insulinotropic polypeptide in the adult rat brain. Journal of neuroscience research. 2007;85(10):2099–119. Epub 2007/05/19. doi: 10.1002/jnr.21349 . 17510976

[35] Usdin TB, Mezey E, Button DC, Brownstein MJ, Bonner TI. Gastric inhibitory polypeptide receptor, a member of the secretin-vasoactive intestinal peptide receptor family, is widely distributed in peripheral organs and the brain. Endocrinology. 1993;133(6):2861–70. Epub 1993/12/01. doi: 10.1210/endo.133.6.8243312 . 8243312

[36] Hamilton A, Patterson S, Porter D, Gault VA, Holscher C. Novel GLP-1 mimetics developed to treat type 2 diabetes promote progenitor cell proliferation in the brain. Journal of neuroscience research. 2011;89(4):481–9. Epub 2011/02/12. doi: 10.1002/jnr.22565 . 21312223

[37] Wang Q, Li L, Xu E, Wong V, Rhodes C, Brubaker PL. Glucagon-like peptide-1 regulates proliferation and apoptosis via activation of protein kinase B in pancreatic INS-1 beta cells. Diabetologia. 2004;47(3):478–87. Epub 2004/02/06. doi: 10.1007/s00125-004-1327-5 . 14762654

[38] Yu YW, Hsueh SC, Lai JH, Chen YH, Kang SJ, Chen KY, et al. Glucose-Dependent Insulinotropic Polypeptide Mitigates 6-OHDA-Induced Behavioral Impairments in Parkinsonian Rats. International journal of molecular sciences. 2018;19(4). Epub 2018/04/12. doi: 10.3390/ijms19041153; PubMed Central PMCID: PMC5979480. 29641447 PMC5979480

[39] Zhou H, Li D, Shi C, Xin T, Yang J, Zhou Y, et al. Effects of Exendin-4 on bone marrow mesenchymal stem cell proliferation, migration and apoptosis in vitro. Scientific reports. 2015;5:12898. Epub 2015/08/08. doi: 10.1038/srep12898; PubMed Central PMCID: PMC4528192. 26250571 PMC4528192

[40] Erbil D, Eren CY, Demirel C, Kucuker MU, Solaroglu I, Eser HY. GLP-1’s role in neuroprotection: a systematic review. Brain injury. 2019;33(6):734–819. Epub 2019/04/03. doi: 10.1080/02699052.2019.1587000 . 30938196

[41] Fontanarosa PB, DeAngelis CD. Translational medical research. Jama. 2003;289(16):2133. Epub 2003/04/24. doi: 10.1001/jama.289.16.2133. 12709473

[42] Woolf SH. The meaning of translational research and why it matters. Jama. 2008;299(2):211–3. Epub 2008/01/10. doi: 10.1001/jama.2007.26 . 18182604

[43] Moher D, Shamseer L, Clarke M, Ghersi D, Liberati A, Petticrew M, et al. Preferred reporting items for systematic review and meta-analysis protocols (PRISMA-P) 2015 statement. Systematic reviews. 2015;4:1. Epub 2015/01/03. doi: 10.1186/2046-4053-4-1; PubMed Central PMCID: PMC4320440. 25554246 PMC4320440

[44] Peters JL, Sutton AJ, Jones DR, Rushton L, Abrams KR. A systematic review of systematic reviews and meta-analyses of animal experiments with guidelines for reporting. Journal of environmental science and health Part B, Pesticides, food contaminants, and agricultural wastes. 2006;41(7):1245–58. Epub 2006/08/23. doi: 10.1080/03601230600857130 . 16923604

[45] Marsili L, Rizzo G, Colosimo C. Diagnostic Criteria for Parkinson’s Disease: From James Parkinson to the Concept of Prodromal Disease. Frontiers in Neurology. 2018;9(156). doi: 10.3389/fneur.2018.00156 29628907 PMC5877503

[46] Goetz CG, Tilley BC, Shaftman SR, Stebbins GT, Fahn S, Martinez-Martin P, et al. Movement Disorder Society-sponsored revision of the Unified Parkinson’s Disease Rating Scale (MDS-UPDRS): scale presentation and clinimetric testing results. Movement disorders: official journal of the Movement Disorder Society. 2008;23(15):2129–70. Epub 2008/11/26. doi: 10.1002/mds.22340 . 19025984

[47] Horváth K, Aschermann Z, Ács P, Deli G, Janszky J, Komoly S, et al. Minimal clinically important difference on the Motor Examination part of MDS-UPDRS. Parkinsonism & related disorders. 2015;21(12):1421–6. Epub 2015/11/19. doi: 10.1016/j.parkreldis.2015.10.006 . 26578041

[48] Chaudhuri KR, Martinez-Martin P. Quantitation of non-motor symptoms in Parkinson’s disease. European journal of neurology. 2008;15 Suppl 2:2–7. Epub 2008/08/22. doi: 10.1111/j.1468-1331.2008.02212.x . 18702736

[49] Goetz CG, Nutt JG, Stebbins GT. The Unified Dyskinesia Rating Scale: Presentation and clinimetric profile. Movement Disorders. 2008;23(16):2398–403. doi: 10.1002/mds.22341 19025759

[50] Peto V, Jenkinson C, Fitzpatrick R, Greenhall R. The development and validation of a short measure of functioning and well being for individuals with Parkinson’s disease. Quality of life research: an international journal of quality of life aspects of treatment, care and rehabilitation. 1995;4(3):241–8. Epub 1995/06/01. doi: 10.1007/bf02260863 . 7613534

[51] de Boer AG, Wijker W, Speelman JD, de Haes JC. Quality of life in patients with Parkinson’s disease: development of a questionnaire. Journal of neurology, neurosurgery, and psychiatry. 1996;61(1):70–4. Epub 1996/07/01. doi: 10.1136/jnnp.61.1.70 ; PubMed Central PMCID: PMC486462. 8676165 PMC486462

[52] Mattis S. Mental status examination for organic mental syndrome in the elderly patient. In: Bellak L, Karasu TB. Geriatric Psychiatry: A Handbook for Psychiatrists and Primary Care Physicians. New York: Grune and Stratton Inc; 1976. pp.77–121. doi: 10.1021/bi00655a025

[53] Montgomery SA, Asberg M. A new depression scale designed to be sensitive to change. The British journal of psychiatry: the journal of mental science. 1979;134:382–9. Epub 1979/04/01. doi: 10.1192/bjp.134.4.382 . 444788

[54] Higgins JPT, Altman DG, Gøtzsche PC, Jüni P, Moher D, Oxman AD, et al. The Cochrane Collaboration’s tool for assessing risk of bias in randomised trials. BMJ. 2011;343:d5928–d. doi: 10.1136/bmj.d5928 . 22008217 PMC3196245

[55] Sterne JAC, Savović J, Page MJ, Elbers RG, Blencowe NS, Boutron I, et al. RoB 2: a revised tool for assessing risk of bias in randomised trials. BMJ. 2019;366:l4898. Epub 2019/08/30. doi: 10.1136/bmj.l4898. 31462531

[56] Hooijmans CR, Rovers MM, de Vries RBM, Leenaars M, Ritskes-Hoitinga M, Langendam MW. SYRCLE’s risk of bias tool for animal studies. BMC Med Res Methodol. 2014;14:43–. doi: 10.1186/1471-2288-14-43 . 24667063 PMC4230647

[57] Sena ES, Currie GL, McCann SK, Macleod MR, Howells DW. Systematic reviews and meta-analysis of preclinical studies: why perform them and how to appraise them critically. J Cereb Blood Flow Metab. 2014;34(5):737–42. Epub 2014/02/19. doi: 10.1038/jcbfm.2014.28 . 24549183 PMC4013765

[58] Egger M, Davey Smith G, Schneider M, Minder C. Bias in meta-analysis detected by a simple, graphical test. BMJ. 1997;315(7109):629–34. Epub 1997/10/06. doi: 10.1136/bmj.315.7109.629 ; PubMed Central PMCID: PMC2127453. 9310563 PMC2127453

[59] Sterne JA, Egger M. Funnel plots for detecting bias in meta-analysis: guidelines on choice of axis. Journal of clinical epidemiology. 2001;54(10):1046–55. Epub 2001/09/29. doi: 10.1016/s0895-4356(01)00377-8 . 11576817

[60] Higgins JPT, Thompson SG, Deeks JJ, Altman DG. Measuring inconsistency in meta-analyses. BMJ. 2003;327(7414):557–60. doi: 10.1136/bmj.327.7414.557 . 12958120 PMC192859

[61] Guyatt GH, Oxman AD, Vist GE, Kunz R, Falck-Ytter Y, Alonso-Coello P, et al. GRADE: an emerging consensus on rating quality of evidence and strength of recommendations. BMJ. 2008;336(7650):924–6. Epub 2008/04/26. doi: 10.1136/bmj.39489.470347.AD ; PubMed Central PMCID: PMC2335261. 18436948 PMC2335261

[62] Hooijmans CR, de Vries RBM, Ritskes-Hoitinga M, Rovers MM, Leeflang MM, IntHout J, et al. Facilitating healthcare decisions by assessing the certainty in the evidence from preclinical animal studies. PloS one. 2018;13(1):e0187271. Epub 2018/01/13. doi: 10.1371/journal.pone.0187271; PubMed Central PMCID: PMC5764235. 29324741 PMC5764235

[63] Wei D, Tang K, Wang Q, Estill J, Yao L, Wang X, et al. The use of GRADE approach in systematic reviews of animal studies. Journal of evidence-based medicine. 2016;9(2):98–104. Epub 2016/03/22. doi: 10.1111/jebm.12198 . 26997212

[64] Guyatt GH, Oxman AD, Vist G, Kunz R, Brozek J, Alonso-Coello P, et al. GRADE guidelines: 4. Rating the quality of evidence—study limitations (risk of bias). Journal of clinical epidemiology. 2011;64(4):407–15. Epub 2011/01/21. doi: 10.1016/j.jclinepi.2010.07.017 . 21247734

[65] Guyatt GH, Oxman AD, Kunz R, Brozek J, Alonso-Coello P, Rind D, et al. GRADE guidelines 6. Rating the quality of evidence—imprecision. Journal of clinical epidemiology. 2011;64(12):1283–93. Epub 2011/08/16. doi: 10.1016/j.jclinepi.2011.01.012 . 21839614

[66] Guyatt GH, Oxman AD, Kunz R, Woodcock J, Brozek J, Helfand M, et al. GRADE guidelines: 7. Rating the quality of evidence—inconsistency. Journal of clinical epidemiology. 2011;64(12):1294–302. Epub 2011/08/02. doi: 10.1016/j.jclinepi.2011.03.017 . 21803546

[67] Guyatt GH, Oxman AD, Kunz R, Woodcock J, Brozek J, Helfand M, et al. GRADE guidelines: 8. Rating the quality of evidence—indirectness. Journal of clinical epidemiology. 2011;64(12):1303–10. Epub 2011/08/02. doi: 10.1016/j.jclinepi.2011.04.014 . 21802903

[68] Guyatt GH, Oxman AD, Montori V, Vist G, Kunz R, Brozek J, et al. GRADE guidelines: 5. Rating the quality of evidence—publication bias. Journal of clinical epidemiology. 2011;64(12):1277–82. Epub 2011/08/02. doi: 10.1016/j.jclinepi.2011.01.011 . 21802904

[69] Tambasco N, Romoli M, Calabresi P. Levodopa in Parkinson’s Disease: Current Status and Future Developments. Curr Neuropharmacol. 2018;16(8):1239–52. doi: 10.2174/1570159X15666170510143821 . 28494719 PMC6187751

[70] Bové J, Perier C. Neurotoxin-based models of Parkinson’s disease. Neuroscience. 2012;211:51–76. Epub 2011/11/24. doi: 10.1016/j.neuroscience.2011.10.057 . 22108613

[71] Prasad EM, Hung S-Y. Behavioral Tests in Neurotoxin-Induced Animal Models of Parkinson’s Disease. Antioxidants (Basel). 2020;9(10):1007. doi: 10.3390/antiox9101007. 33081318 PMC7602991

