# Supplementary material for: GLP-1 and GIP receptor agonists in the treatment of Parkinson’s disease: Translational systematic review and meta-analysis protocol of clinical and preclinical studies
Source: PLoS One. 2021 Aug 12;16(8):e0255726. doi: 10.1371/journal.pone.0255726 (PMC8360366; doi:10.1371/journal.pone.0255726)
Supplement: S1 Appendix — Search strategy example to be adapted for each database. (DOCX) [file pone.0255726.s002.docx]

**S1 Appendix. Search Strategy – OVID MEDLINE.** Search strategy example to be adapted for each database.

| 1. | Parkinson Disease.mp. or exp Parkinson Disease/ |
| --- | --- |
| 2. | Idiopathic Parkinson's Disease.mp. |
| 3. | Lewy Body Parkinson Disease.mp. |
| 4. | Lewy Body Parkinson's Disease.mp. |
| 5. | Primary Parkinsonism.mp. |
| 6. | Parkinsonism, Primary.mp. |
| 7. | Parkinson's disease.mp. |
| 8. | Parkinson's Disease, Idiopathic.mp. |
| 9. | Parkinson's Disease, Lewy Body.mp. |
| 10. | Idiopathic Parkinson Disease.mp. |
| 11. | Paralysis Agitans.mp. |
| 12. | Parkinsonian Disorders.mp. or exp Parkinsonian Disorders/ |
| 13. | Parkinsonian Syndrome.mp. |
| 14. | Parkinsonism.mp. |
| 15. | Parkinsonian Syndromes.mp. |
| 16. | Parkinsonian Diseases.mp. |
| 17. | Autosomal Dominant Parkinsonism.mp. |
| 18. | Autosomal Recessive Juvenile Parkinson Disease.mp. |
| 19. | Chromosome 6-Linked Autosomal Recessive Parkinsonism.mp. |
| 20. | Chromosome 6 Linked Autosomal Recessive Parkinsonism.mp. |
| 21. | Parkinsonism, Juvenile, Autosomal Recessive.mp. |
| 22. | Parkinson Disease Autosomal Recessive, Early Onset.mp. |
| 23. | Familial Juvenile Parkinsonism.mp. |
| 24. | Parkinsonism, Experimental.mp. |
| 25. | Experimental Parkinsonisms.mp. |
| 26. | Parkinson Disease, Experimental.mp. |
| 27. | MPTP-Induced Experimental Parkinsonism.mp. |
| 28. | MPTP Induced Experimental Parkinsonism.mp. |
| 29. | Experimental Parkinson Disease.mp. |
| 30. | Experimental Parkinsonism.mp. |
| 31. | Parkinsonism, Juvenile.mp. |
| 32. | Juvenile Parkinsonism.mp. |
| 33. | Juvenile Parkinson Disease.mp. |
| 34. | Autosomal Recessive Parkinsonism.mp. |
| 35. | Parkinsonism, Autosomal Recessive.mp. |
| 36. | exp Glucagon-Like Peptide 1/ or Glucagon-Like Peptide 1.mp. |
| 37. | Glucagon Like Peptide 1.mp. |
| 38. | GLP-1.mp. |
| 39. | GLP 1.mp. |
| 40. | Glucagon-Like Peptide-1.mp. |
| 41. | Exenatide.mp. or exp Exenatide/ |
| 42. | Bydureon.mp. |
| 43. | ITCA 650.mp. |
| 44. | AC 2993 LAR.mp. |
| 45. | Exendin-4.mp. |
| 46. | Ex4 Peptide.mp. |
| 47. | Exendin 4.mp. |
| 48. | Byetta.mp. |
| 49. | AC 2993.mp. |
| 50. | liraglutide.mp. or exp Liraglutide/ |
| 51. | Victoza.mp. |
| 52. | Saxenda.mp. |
| 53. | NN 2211.mp. |
| 54. | 2211, NN.mp. |
| 55. | NN2211.mp. |
| 56. | NN-2211.mp. |
| 57. | lixisenatide.mp. |
| 58. | Adlyxin.mp. |
| 59. | ZP10A peptide.mp. |
| 60. | ZP 10.mp. |
| 61. | ZP-10.mp. |
| 62. | Lyxumia.mp. |
| 63. | "AVE 0010".mp. |
| 64. | AVE0010.mp. |
| 65. | Gastric Inhibitory Polypeptide.mp. or exp Gastric Inhibitory Polypeptide/ |
| 66. | Inhibitory Polypeptide, Gastric.mp. |
| 67. | Polypeptide, Gastric Inhibitory.mp. |
| 68. | Glucose-Dependent Insulin-Releasing Peptide.mp. |
| 69. | Glucose Dependent Insulin Releasing Peptide.mp. |
| 70. | Glucose Dependent Insulinotropic Peptide.mp. |
| 71. | Glucose-Dependent Insulinotropic Peptide.mp. |
| 72. | Insulinotropic Peptide, Glucose-Dependent.mp. |
| 73. | Peptide, Glucose-Dependent Insulinotropic.mp. |
| 74. | Gastric-Inhibitory Polypeptide.mp. |
| 75. | Polypeptide, Gastric-Inhibitory.mp. |
| 76. | GIP.mp. |
| 77. | Semaglutide.mp. |
| 78. | Glucagon-Like Peptide-1 Receptor.mp. or exp Glucagon-Like Peptide-1 Receptor/ |
| 79. | Glucagon Like Peptide 1 Receptor.mp. |
| 80. | Receptor, Glucagon-Like Peptide-1.mp. |
| 81. | GLP-1R Receptor.mp. |
| 82. | GLP 1R Receptor.mp. |
| 83. | Receptor, GLP-1R.mp. |
| 84. | GLP1R Protein.mp. |
| 85. | GLP-1 Receptor.mp. |
| 86. | GLP 1 Receptor.mp. |
| 87. | Receptor, GLP-1.mp. |
| 88. | Receptor, GLP1R.mp. |
| 89. | Geniposide.mp. |
| 90. | Jasminoidin.mp. |
| 91. | D-Ala2-GIP-glu-PAL.mp. |
| 92. | Ala2-GIP-Glu-PAL.mp. |
| 93. | DA-JC1.mp. |
| 94. | DA-JC4.mp. |
| 95. | DA-CH5.mp. |
| 96. | DA3-CH.mp. |
| 97 | Oxytomodulin.mp. |
| 98. | 1 or 2 or 3 or 4 or 5 or 6 or 7 or 8 or 9 or 10 or 11 or 12 or 13 or 14 or 15 or 16 or 17 or 18 or 19 or 20 or 21 or 22 or 23 or 24 or 25 or 26 or 27 or 28 or 29 or 30 or 31 or 32 or 33 or 34 or 35 |
| 99. | 36 or 37 or 38 or 39 or 40 or 41 or 42 or 43 or 44 or 45 or 46 or 47 or 48 or 49 or 50 or 51 or 52 or 53 or 54 or 55 or 56 or 57 or 58 or 59 or 60 or 61 or 62 or 63 or 64 or 65 or 66 or 67 or 68 or 69 or 70 or 71 or 72 or 73 or 74 or 75 or 76 or 77 or 78 or 79 or 80 or 81 or 82 or 83 or 84 or 85 or 86 or 87 or 88 or 89 or 90 or 91 or 92 or 93 or 94 or 95 or 96 or 97 |
| 100. | 98 and 99 |
